# Risk prediction score and equation for progression of arterial stiffness using Japanese longitudinal health examination data

**DOI:** 10.1038/s41440-024-02057-z

**Published:** 2025-02-20

**Authors:** Naoko Inadome, Shin Kawasoe, Masaaki Miyata, Takuro Kubozono, Satoko Ojima, Ryuko Mori, Hironori Miyahara, Koichi Tokushige, Mitsuru Ohishi

**Affiliations:** 1 Graduate School of Health Sciences, Faculty of Medicine, Kagoshima University, Kagoshima, Japan; 2 Department of Nursing, Faculty of Nursing, International University of Kagoshima, Kagoshima, Japan; 3 Department of Cardiovascular Medicine and Hypertension, Graduate School of Medical and Dental Sciences, Kagoshima University, Kagoshima, Japan; 4 International Center for Island Studies, Kagoshima University, Kagoshima, Japan; 5 Kagoshima Kouseiren Hospital, Kagoshima, Japan

**Keywords:** Arterial stiffness, baPWV, Prediction equation, Prediction score

## Abstract

The brachial-ankle pulse wave velocity (baPWV) is useful for evaluating arterial stiffness. No longitudinal studies have examined the association between multiple arterial stiffness risk factors and increased baPWV. We sought to identify factors associated with baPWV ≥1400 cm/s within 5 years and create an equation and simple risk score to predict its occurrence, using data from a large-scale Japanese health examination database. Of 10,284 participants aged 30–69 years for whom follow-up data were available over a 5-year period, 3394 men and 2710 women with baseline baPWV<1400 cm/s were analyzed. We used age, body mass index (BMI), systolic blood pressure (SBP), diastolic blood pressure (DBP), heart rate (HR), fasting blood sugar (FBS), low-density lipoprotein cholesterol (LDL-C), high-density lipoprotein cholesterol (HDL-C), triglyceride (TG), estimated glomerular filtration rate (eGFR), habitual exercise, habitual drinking, and smoking history as variables. In the multivariate logistic regression analysis, baPWV≥1400 cm/s was associated significantly with age, BMI, SBP, DBP, HR, FBS, and TG in men and age, SBP, DBP, HR, and smoking history in women. A prediction score based on these factors yielded an area under the curve (AUC) for the 5-year incidence of baPWV≥1400 cm/s of 0.68 for men and 0.71 for women. Furthermore, a risk prediction equation for the 5-year incidence of baPWV≥1400 cm/s showed an AUC = 0.71 for men and 0.77 for women. The prediction equation and a simple prediction score are easy to implement clinically. The predictive ability of these scores and equations for arterial stiffness should be validated in prospective studies.

**Figure:**
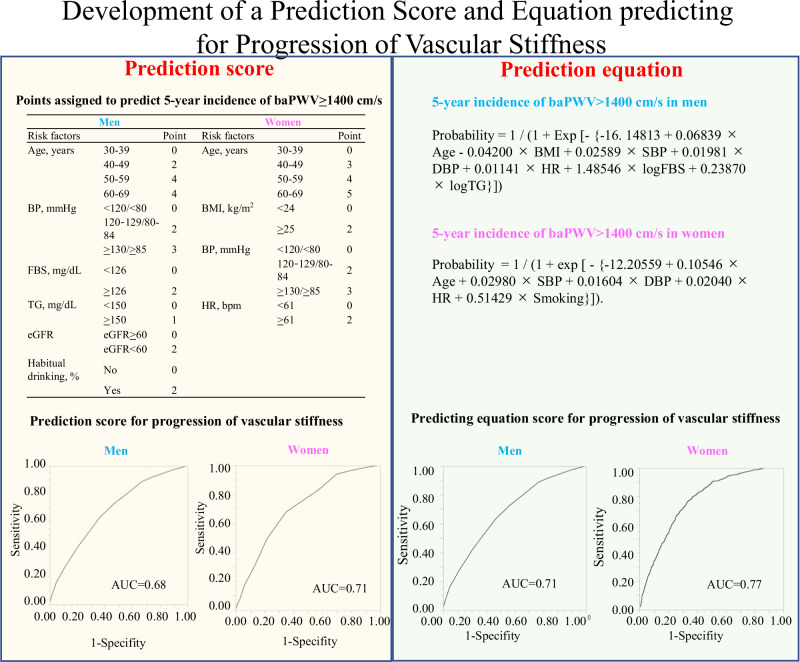
The risk score is the sum of all points, which risk factors were significantly associated with the 5-year incidence of baPWV>1400 cm/s in the multivariate logistic regression analysis. The AUC for the 5-year incidence of baPWV>1400 cm/s was 0.68 for men and 0.71 in women.

The risk score is the sum of all points, which risk factors were significantly associated with the 5-year incidence of baPWV>1400 cm/s in the multivariate logistic regression analysis. The AUC for the 5-year incidence of baPWV>1400 cm/s was 0.68 for men and 0.71 in women.

## Abstract (web summary)

The risk score is the sum of all points, which risk factors were significantly associated with the 5-year incidence of baPWV≥1400 cm/s in the multivariate logistic regression analysis. The AUC for the 5-year incidence of baPWV≥1400 cm/s was 0.68 for men and 0.71 in women.

## Introduction

Increased arterial stiffness is a pathology of vascular disorders and is associated with atherosclerosis [1, 2]. Early detection and prevention of the onset and progression of arterial stiffness are crucial. Risk factors for arterial stiffness include hypertension, dyslipidemia, diabetes mellitus (DM), chronic kidney disease (CKD), heart rate (HR), smoking, age, male sex, and obesity [3–10].

Brachial-ankle pulse wave velocity (baPWV) has been reported to be a useful index for evaluating arterial stiffness [11]. baPWV is an established and relatively simple index for measuring pulse wave velocity (PWV). Several studies have investigated the association between baPWV and risk factors for arterial stiffness. Cross-sectional studies have reported associations of elevated baPWV with hypertension, HR, diabetes mellitus, dyslipidemia, obesity, estimated glomerular filtration rate (eGFR), smoking, alcohol consumption, and metabolic syndrome [10, 12–14]. Longitudinal studies have also reported associations between an elevated baPWV and hypertension, HR, triglyceride (TG) levels, eGFR, alcohol consumption, and smoking. However, these longitudinal studies have only examined the association between limited factors and increased baPWV [6, 15–17]. Furthermore, no longitudinal studies have examined the association between multiple risk factors of arterial stiffness and increased baPWV.

In Japan, since 2008, specific health medical examinations have been recommended for people aged 40 years and older to prevent lifestyle-related diseases, and specific health guidance has been provided based on the results of these specific health medical examinations. Predicting the progression of atherosclerosis using data from specific medical examinations would be clinically useful. We have previously conducted several longitudinal studies using data obtained from large-scale health examinations in Japan. We then developed simple risk scores and equations to predict the development of hypertension, CKD, and metabolic syndrome and reported their validity [18–20]. However, we did not identify factors associated with elevated baPWV in these previous studies.

Therefore, this study aimed to identify factors associated with baPWV ≥ 1400 cm/s after 5 years and to create an equation and simple risk score to predict a baPWV ≥ 1400 cm/s after 5 years, using medical interview data, blood test results, and anthropometric measurements obtained from large-scale Japanese health examinations.

Point of view

- **Clinical relevance**
  This study showed that age, BP, FBS, TG, eGFR, and habitual drinking were significantly associated with the 5-year incidence of baPWV ≥ 1400 cm/s in men, whereas age, BMI, BP, and HR were significantly associated with it in women.
- **Future direction**
  Although we developed a risk prediction score and risk prediction equation for arterial stiffness in each sex using above factors, the predictive ability of these score and equation should be validated in future prospective studies.
- **Considerations for the Asian Population**
  These risk score and equation to predict baPWV ≥ 1400 cm/s after 5 years may be useful for early lifestyle modifications and mediations to prevent arterial stiffness among Asians.

## Methods

### Data sources and study participants

Data were collected from 11,185 participants who underwent at least two physical examinations at Kagoshima Kouseiren Hospital between April 2005 and March 2019. Participants aged 30–69 years for whom data were available at baseline and 5 years later (range: 3–7 years) and who had undergone baPWV testing on both occasions were selected. The mean duration was 4.8 ± 0.7 years, with 71.8% of subjects having a duration between 4.5 and 5.5 years. We also excluded participants with missing data (*N* = 24), ankle-brachial index (ABI) < 0.9 (*N* = 20), atrial fibrillation (AF) (*N* = 32), and TG ≥ 400 mg/dL (*N* = 135) and those on medication for hypertension, DM, or dyslipidemia (*N* = 690). Thus, we included 10,284 participants aged 30–69 years for whom follow-up data were available at 5 years. Of these, 6104 (men = 3394, women = 2710) with baPWV < 1400 cm/s at baseline were included in this study.

This study was conducted in accordance with the Declaration of Helsinki and was approved by the ethical review committee of the Graduate School of Medical and Dental Sciences, Kagoshima University. Informed consent was obtained by opt-out because only existing anonymized data were used in this study.

### Candidate risk factors

Patients were classified into four age groups: 30–39, 40–49, 50–59, and 60–69 years. Trained personnel measured height and weight using standard anthropometric methods. Body mass index (BMI) was calculated as weight (kg) divided by the square of height (m^2^) and was classified into two groups: < 25 kg/m^2^ and ≥ 25 kg/m^2^. Blood pressure (BP) was measured after sitting for 5 min. BP was classified into three groups with reference to the Japanese Society of Hypertension Guidelines for the Management of Hypertension (JSH 2019): normal, < 120/ < 80 mmHg; normal–high, 120–129/80–84 mmHg, and high, ≥ 130/ ≥ 85 mmHg [21]. The HR was classified into two groups according to the median value: HR < 61 bpm and HR ≥ 61 bpm. Blood samples were collected after overnight fasting for measurement of fasting blood sugar (FBS), low-density lipoprotein cholesterol (LDL-C), high-density lipoprotein cholesterol (HDL-C), and TG levels. FBS was classified into two groups according to the Japanese Clinical Practice Guidelines for Diabetes 2019: FBS < 126 mg/dL and FBS ≥ 126 mg/dL, according to the diagnostic criteria of the Japan Diabetes Society Diabetes Guidelines 2019 [22]. Dyslipidemia was classified into two groups according to the Japan Atherosclerosis Society Guidelines for Prevention of Atherosclerotic Cardiovascular Diseases 2022: HDL-C was classified into HDL-C ≥ 40 mg/dL, HDL-C < 40 mg/dL, LDL-C into LDL-C < 140 mg/dL, and LDL-C ≥ 140 mg/dL, and TG was classified into TG < 150 mg/dL and TG ≥ 150 mg/dL [23]. The eGFR was determined according to the new Japanese coefficient for the modified isotope dilution mass spectrometry-traceable Modification of Diet in Renal Disease study equation: eGFR = 194 × SCr^−1.094^ × Age^−0.287^, where SCr represents serum creatinine levels (mg/dL). For women, eGFR was multiplied by a correction factor of 0.739 [24]. eGFR was classified into two groups eGFR ≥ 60 mL/min/1.73 m^2^ and < 60 mL/min/1.73 m^2^. Data on medications for hypertension, diabetes mellitus, and dyslipidemia and information on habitual exercise, habitual drinking, and smoking history were obtained using a self-administered questionnaire. Habitual exercise was defined as a sweat-inducing exercise of at least 30 min per session performed at least twice a week for at least one year. Alcohol consumption was defined as habitual drinking for > 10 days/month. Patients were classified as current smokers or nonsmokers, including those with no smoking history and past smokers.

baPWV was measured using a Colin Waveform Analyzer (Colin, Komaki, Japan), as previously reported [11, 12]. Briefly, pulse wave recording (PVR) was performed at a cuff pressure of 60 mmHg. The baPWV was automatically calculated according to the following equation: baPWV (cm/s) = (D1–D2) / T, where D1 is the distance from the heart to the right ankle, D2 is the distance from the heart to the right upper arm, and T is the time from onset of the increase in the PVR at the right upper arm to the onset of the increase in the PVR at the right ankle. The distance was automatically calculated based on each participant’s height. The mean baPWV was calculated as the average of the right and left baPWV values. The outcome was defined as a baPWV ≥ 1400 cm/s at the 5-year follow-up.

### Statistical analysis

All analyses were performed separately for men and women. Continuous variables, including age, BMI, systolic blood pressure (SBP), diastolic blood pressure (DBP), HR, LDL-C, HDL-C, and eGFR, are expressed as mean ± standard deviation. Values with skewed distribution, including FBS and TG, are expressed as medians [1st quartile, 3rd quartile]. Categorical variables, such as habitual exercise, habitual drinking, and smoking history, are expressed as numbers and percentages. The incidence of baPWV ≥ 1400 cm/s after 5 years was also calculated.

Univariate and multivariate logistic regression analyses were also performed. The odds ratios and 95% confidence intervals for the incidence of baPWV ≥ 1400 cm/s for each variable were calculated. In the multivariate logistic regression analyses, the odds ratios for the 5-year incidence of baPWV ≥1400 cm/s were adjusted for age, BMI, SBP, DBP, HR, log FBS, LDL-C, HDL-C, log TG, eGFR, habitual exercise, habitual drinking, and smoking history.

A risk score was then created to predict the 5-year incidence of baPWV ≥ 1400 cm/s. The following points corresponding to standardized beta coefficients were assigned to each risk factor category based on the methodology used by the Japan Epidemiology Collaboration on Occupational Health Study Group: 1, β = 0.01–0.20; 2, β = 0.21–0.80; 3, β = 0.81–1.20; 4, β = 1.21–2.20; and 5, β > 2.20. The reference for each variable was given a point of 0. The risk score for the 5-year incidence of baPWV ≥ 1400 cm/s was calculated as the sum of the individual points. We then used the β coefficients for the risk factors that were significant in the logistic regression analysis to create an equation that directly calculates the proportion of the 5-year incidence of baPWV ≥ 1400 cm/s. In developing the equation, continuous variables such as age, BMI, SBP, DBP, HR, FBS, and TG were used as continuous values without categorization.

The diagnostic performance of the risk score and the equation were evaluated using the area under the curve (AUC) from receiver operating characteristic curve (ROC) analysis. The Youden index was calculated based on the sensitivity and specificity of each score. The maximum score was used as the cut-off value.

All statistical analyses were performed using JMP Pro version 15 (SAS Institute, Cary, NC, USA) for Windows. *P* value < 0.05 was considered significant.

## Results

### Characteristics of study participants

Overall, 5-year follow-up data were available for 10,284 participants aged 30–69 years. Of these, 6104 (3394 men, 2710 women) with a baseline baPWV < 1400 cm/s were included in the analysis. Table 1 presents the baseline characteristics of the study participants. The mean age of men and women was 47.1 ± 8.6 years and 48.8 ± 9.0 years, respectively. The mean baPWV was 1261.0 ± 90.4 cm/s for men and 1214.1 ± 109.1 cm/s for women.

**Table 1:** Characteristics of study population with baPWV <1400 cm/s at baseline

	Men (*n* = 3394)	Women (*n* = 2710)
Age, years	47.1 ± 8.6	48.8 ± 9.0
BMI, kg/m^2^	23.6 ± 3.1	22.3 ± 3.2
SBP, mmHg	114.0 ± 12.6	108.4 ± 13.5
DBP, mmHg	74.4 ± 9.8	69.1 ± 9.4
HR, bpm	60.1 ± 8.3	63.1 ± 8.7
Mean baPWV, cm/s	1261.0 ± 90.4	1214.1 ± 109.1
FBS, mg/dL	99 [94, 106]	95 [90, 100]
LDL-C, mg/dL	127.6 ± 32.0	124.6 ± 31.6
HDL-C, mg/dL	56.1 ± 13.7	66.4 ± 14.2
TG, mg/dL	105 [73, 157]	70 [52,94]
eGFR, mL/min/1.73 m^2^	82.4 ± 13.5	83.5 ± 14.7
Habitual exercise, %	30.6	34.5
Habitual drinking, %	60.2	17.4
Smoking history, %	43.9	5.8

*BMI* body mass index, *SBP* systolic blood pressure, *DBP* diastolic blood pressure, *HR* Heart rate, *Mean baPWV* mean brachial-ankle pulse wave velocity, *FBS* fasting blood sugar, *LDL-C* low-density lipoprotein cholesterol, *HDL-C* high-density lipoprotein cholesterol, *TG* triglycetlides, *eGFR* estimated glomerular filtration rate, Continuous variables are expressed as mean ± standard deviation, except for TG and FBS, which are expressed as median [1st quartile, 3rd quartile]

### Logistic regression analysis of each risk factor for the 5-year incidence of baPWV ≥ 1400 cm/s

Of the total participants, 1107 (32.6%) of 3394 men and 671 (24.8%) of 2710 women developed baPWV ≥ 1400 cm/s after 5 years. The results of the logistic regression analysis of each risk factor for the 5-year incidence of baPWV ≥ 1400 cm/s are shown in Table 2. Univariate and multivariate logistic regression analyses were performed separately for men and women. Univariate logistic regression analysis showed that age, SBP, DBP, HR, FBS, TG, eGFR, habitual drinking, and smoking history were significantly associated with the 5-year incidence of baPWV ≥ 1400 cm/s in men. In women, age, BMI, SBP, DBP, FBS, LDL-C, HDL-C, TG, and eGFR were significantly associated with a 5-year incidence of baPWV ≥ 1400 cm/s. In the multivariate logistic regression analysis, age, BMI, SBP, DBP, HR, FBS, and TG were significantly associated with the 5-year incidence of baPWV ≥ 1400 cm/s in men, and age, SBP, DBP, HR, and smoking history were significantly associated with the 5-year incidence of baPWV ≥ 1400 cm/s in women.

**Table 2:** Logistic regression analysis of each risk factor for the 5-year incidence of baPWV ≥ 1400 cm/s

Risk factors	Men				Women				
	univaliable		multivaliable			univaliable		multivaliable	
	OR (95% CI)	*P* value	OR (95%CI)	*P* value		OR (95% CI)	*P* value	OR (95% CI)	*P* value
Age, years	1.07 (1.06–1.08)	< 0.0001	1.07 (1.06–1.08)	<0.0001		1.11 (1.09–1.12)	< 0.0001	1.11 (1.10–1.13)	< 0.0001
BMI, kg/m^2^	1.02 (1.00–1.04)	0.0962	0.96 (0.93–0.99)	0.0080		1.09 (1.06–1.12)	< 0.0001	1.00 (0.97–1.04)	0.9693
SBP, mmHg	1.04 (1.03–1.04)	< 0.0001	1.03 (1.02–1.03)	< 0.0001		1.04 (1.04–1.05)	< 0.0001	1.03 (1.02–1.04)	< 0.0001
DBP, mmHg	1.04 (1.04–1.05)	< 0.0001	1.02 (1.01–1.03)	0.0006		1.05 (1.04–1.06)	< 0.0001	1.02 (1.00–1.03)	0.0330
HR, bpm	1.01 (1.00–1.02)	0.0097	1.01 (1.00–1.02)	0.0147		1,00 (0.99–1.01)	0.9443	1.02 (1.00–1.03)	0.0070
logFBS*	10.91 (6.10–19.53)	< 0.0001	4.60 (2.48–8.56)	< 0.0001		27.40 (10.85–69.22)	< 0.0001	2.72 (0.98–7.54)	0.0540
LDL-C, mg/dL	1.00 (1.00–1.00)	0.1385	1.00 (1.00–1.00)	0.7308		1.01 (1.01–1.01)	< 0.0001	1.00 (1.00–1.00)	0.9814
HDL-C, mg/dL	1.00 (0.99–1.00)	0.5098	1.00 (0.99–1.00)	0.4067		0.99 (0.98–0.99)	< 0.0001	0.99 (0.99–1.00)	0.1384
logTG*	1.23 (1.08–1.40)	0.0025	1.21 (1.03–1.44)	0.0245		1.91 (1.58–2.31)	< 0.0001	1.04 (0.81–1.34)	0.7406
eGFR, mL/min/1.73 m^2^	0.99 (0.98–1.00)	0.0003	1.00 (0.99–1.01)	0.8482		0.99 (0.98–0.99)	< 0.0001	1.00 (1.00–1.01)	0.2658
Habitual exercise	1.15 (0.98–1.34)	0.0836	1.05 (0.89–1.25)	0.5604		1.19 (1.00–1.43)	0.0523	0.90 (0.73–1.10)	0.2958
Habitual drinking	1.48 (1.27–1.71)	< 0.0001	1.16 (0.98–1.37)	0.0923		0.94 (0.74–1.18)	0.5874	0.94 (0.72–1.23)	0.6455
Smoking history	0.78 (0.67–0.90)	0.0006	1.15 (0.97–1.36)	0.1021		0.82 (0.56–1.22)	0.3303	1.67 (1.08–2.58)	0.0220

*BMI* body mass index, *SBP* systolic blood pressure, *DBP* diastolic blood pressure, *HR* Heart rate, *Mean baPWV* mean brachial-ankle pulse wave velocity, *FBS* fasting blood sugar, *LDL-C* low-density lipoprotein cholesterol, *HDL-C* high-density lipoprotein cholesterol, *TG* riglycetides, *eGFR* estimated glomerular filtration rate, *OR* odds ratio, *CI* confidence interval, * FBS (mg/dl) and TG (mg/dl) were log transformed. The odds ratios and 95% confidence intervals of 5-year baPWV incidence for each risk factor using logistic regression analysis. In multivariable model, the odds ratios were adjusted for the following variables: age, BMI, SBP, DBP, HR, logFBS, LDL-C, HDL-C, logTG, habitual exercise, habitual drinking, and smoking history

Each risk score was then categorized, and baPWV ≥ 1400 cm/s and its association with each category are shown in Table 3. We performed univariate and multivariate logistic regression analyses separately for men and women. In men, older age, higher BP, higher HR, higher FBS, higher TG, lower eGFR, habitual drinking, and smoking history were significantly associated with the 5-year incidence of baPWV ≥ 1400 cm/s in univariate logistic regression analysis, and older age, higher BP, higher FBS, higher TG, lower eGFR, and habitual drinking were significantly associated with the 5-year incidence of baPWV ≥ 1400 cm/s in multivariate logistic regression analysis. Furthermore, in women, in univariate logistic regression analysis, older age, higher BMI, higher BP, higher HR, and higher LDL-C were significantly associated with the 5-year incidence of baPWV ≥ 1400 cm/s. In multivariable logistic regression analysis, older age, higher BMI, higher BP, and higher HR were associated with the 5-year incidence of baPWV ≥ 1400 cm/s in women.

**Table 3:** Logistic regression analysis of category each risk factor for the 5-year incidence of baPWV ≥ 1400 cm/s

Risk factors		Men					
		No. of Subjects	No. of cases (%)	univaliable		multivaliable	
				OR (95%CI)	*P* value	OR (95%CI)	*P* value
Age, years	30–39	736	123 (16.7)	ref		ref	
	40–49	1355	389 (28.7)	2.01 (1.60–2.52)	< 0.0001	1.72 (1.36–2.17)	< 0.0001
	50–59	995	432 (43.4)	3.82 (3.04–4.82)	< 0.0001	3.43 (2.70–4.37)	< 0.0001
	60–69	308	163 (52.9)	5.60 (4.17–7.53)	< 0.0001	5.38 (3.92–7.36)	< 0.0001
BMI, kg/m^2^	<25	2,374	766 (32.3)	ref		ref	
	≥25	1020	341 (33.4)	1.05 (0.81–1.11)	0.5068	0.89 (0.75–1.07)	0.2056
BP, mmHg	<120/ < 80	1988	503 (25.3)	ref		ref	
	120–129/80–84	869	353 (40.5)	2.01 (1.70–2.38)	< 0.0001	1.91 (1.59–2.28)	< 0.0001
	≥130/ ≥ 85	537	252 (46.9)	2.61 (2.14–3.18)	< 0.0001	2.52 (2.04–3.12)	< 0.0001
HR, bpm	<61	1845	581 (31.5)	ref		ref	
	≥61	1549	526 (34.0)	1.12 (0.96–1.29)	< 0.0001	1.16 (0.99–1.36)	0.0586
FBS, mg/dL	FBS < 126	3247	1028 (31.6)	ref		ref	
	FBS ≥ 126	147	79 (53.7)	2.51 (1.80–3.50)	< 0.0001	2.14 (1.50–3.04)	< 0.0001
LDL-C, mg/dL	LDL-C < 140	2293	730 (31.8)	ref		ref	
	LDL-C ≥ 140	1101	377 (32.4)	1.11 (0.96–1.30)	0.1618	1.04 (0.88–1.23)	0.6452
HDL-C, mg/dL	HDL-C ≥ 40	3099	1007 (32.5)	ref		ref	
	HDL-C < 40	295	100 (33.9)	1.07 (0.83–1.37)	0.6231	1.15 (0.87–1.53)	0.3200
TG, mg/dL	TG < 150	2459	775 (31.5)	ref		ref	
	TG ≥ 150	935	332 (35.5)	1.20 (1.02–1.40)	0.0241	1.21 (1.01–1.45)	0.0374
eGFR, mL/min/1.73 m^2^	eGFR≥60	3286	1049 (31.9)	ref		ref	
	eGFR<60	108	58 (53.7)	1.20 (1.02–1.40)	0.0268	1.59 (1.05–2.41)	0.0275
Habitual exercise	No	2354	746 (31.7)	ref		ref	
	Yes	1040	361 (34.7)	0.87 (0.98–1.33)	0.0836	1.02 (0.86–1.20)	0.8283
Habitual drinking	No	1350	372 (27.6)	ref		ref	
	Yes	2044	735 (36.0)	1.48 (1.27–1.71)	< 0.0001	1.27 (1.07–1.49)	0.0047
Smoking history	No	1903	667 (35.1)	ref		ref	
	Yes	1491	440 (29.5)	0.78 (0.67–0.90)	0.0006	1.04 (0.88–1.22)	0.6644

Risk factors		Women					
		No. of Subjects	No. of cases (%)	univaliable		multivaliable	
				OR (95%CI)	*P* value	OR (95%CI)	*P* value
Age, years	30–39	507	34 (6.7)	ref		ref	
	40–49	887	136 (15.3)	2.51 (1.70–3.73)	< 0.0001	2.33 (1.56–3.47)	< 0.0001
	50–59	953	309 (32.4)	6.68 (4.60–9.70)	< 0.0001	5.97 (4.05–8.79)	< 0.0001
	60–69	363	192 (52.9)	15.6 (10.42–23.41)	< 0.0001	15.69 (10.20–24.00)	< 0.0001
BMI, kg/m^2^	<25	2230	506 (22.7)	ref		ref	
	≥25	480	165 (34.4)	1.78 (1.44–2.21)	< 0.0001	1.41 (1.11–1.79)	0.0055
BP, mmHg	<120/ < 80	2043	408 (20.0)	ref		ref	
	120–129/80–84	445	168 (37.6)	2.43 (1.95–3.03)	< 0.0001	2.13 (1.68–2.71)	< 0.0001
	≥130/ ≥ 85	222	95 (42.8)	3.00 (2.25–4.00)	< 0.0001	2.37 (1.74–3.25)	< 0.0001
HR, bpm	<61	1108	272 (24.6)	ref		ref	
	≥61	1602	339 (24.9)	1.02 (0.85–1.22)	< 0.0001	1.33 (1.90–1.62)	0.0052
FBS, mg/dL	FBS < 126	2670	653 (24.5)	ref		ref	
	FBS ≥ 126	40	18 (45.0)	1.28 (0.87–1.89)	0.2078	1.42 (0.72–2.80)	0.3139
LDL-C, mg/dL	LDL-C < 140	1937	416 (21.5)	ref		ref	
	LDL-C ≥ 140	773	255 (33.0)	1.80 (1.49–2.17)	< 0.0001	1.12 (0.91–1.38)	0.2719
HDL-C, mg/dL	HDL-C ≥ 40	2662	657 (24.7)	ref		ref	
	HDL-C < 40	48	14 (29.2)	1.26 (0.67–2.36)	0.4763	0.83 (0.48–1.96)	0.9367
TG, mg/dL	TG < 150	2540	622 (24.5)	ref		ref	
	TG ≥ 150	170	49 (28.8)	1.25 (0.89–1.77)	0.2004	0.83 (0.57–1.23)	0.3560
eGFR, mL/min/1.73 m^2^	eGFR≥60	2618	638 (24.4)	ref		ref	
	eGFR<60	92	33 (35.9)	1.25 (0.89–1.76)	0.2056	1.25 (0.78–2.01)	0.3504
Habitual exercise	No	1776	419 (23.6)	ref		ref	
	Yes	934	252 (27.0)	0.836 (0.70–1.00)	0.0523	0.93 (0.76–1.14)	0.4978
Habitual drinking	No	2239	559 (25.0)	ref		ref	
	Yes	471	112 (23.8)	0.94 (0.74–1.18)	0.5874	0.97 (0.75–1.26)	0.9715
Smoking history	No	2552	637 (25.0)	ref		ref	
	Yes	158	34 (21.5)	0.82 (0.56–1.22)	0.3314	1.44 (0.94–2.20)	0.0914

*BMI* body mass index, *BP* blood pressure, *HR* Heart rate, *FBS* fasting blood sugar, *LDL-C* low-density lipoprotein cholesterol, *HDL-C* high-density lipoprotein cholesterol, *TG* triglycerides, *eGFR* estimated glomerular filtration rate, *OR* odds ratio, *CI* confidence interval. The odds ratios and 95% confidence intervals of 5-year baPWV incidence of each risk factor using logistic regression analysis. In the multivariable model, the odds ratios were adjusted for the following variables: age, BMI, BP, HR, FBS, LDL-C, HDH-C, TG and eGFR categories, habitual exercise, habitual drinking, and smoking history

### Development of a prediction score for the progression of vascular stiffness

**Fig. 1:**
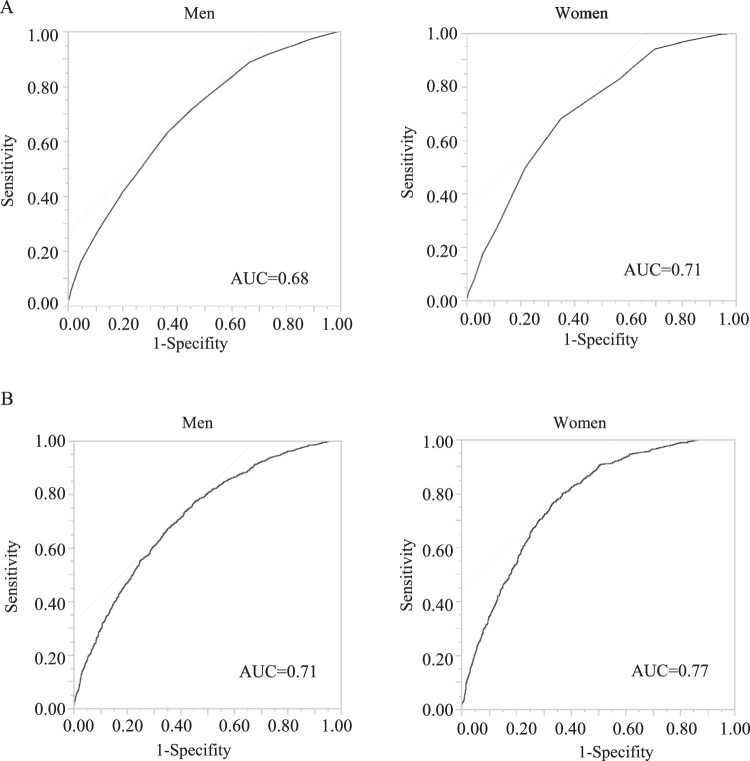
ROC for the 5-year incidence of baPWV ≥1400 cm/s. **A** ROC curves of the risk-prediction score in men and women; **B** ROC curves plotted using continuous values of the risk factors of the prediction equation in men and women. ROC receiver operating characteristic, AUC area under the curve

The points assigned to predict the 5-year incidence of baPWV ≥ 1400 cm/s are shown in Table 4. The risk score was determined as the sum of the scores of risk factors that were significantly associated with the 5-year incidence of baPWV ≥ 1400 cm/s in multivariable logistic regression analysis (Table 3). The risk scores for men and women ranged from 0 to 14 and 0 to 12, respectively. The ability of the risk score to predict baPWV ≥ 1400 cm/s was assessed based on the AUC in the ROC analysis. The AUC for the 5-year incidence of baPWV ≥ 1400 cm/s was 0.68 for men and 0.71 for women (Fig. 1A). The AUCs were higher in women than in men.

**Table 4:** Points assigned to predict 5-year incidence of baPWV ≥ 1400 cm/s

Men				Women			
Risk factors		β	Point	Risk factors		β	Point
Age, years	30–39		0	Age, years	30–39		0
	40–49	0.54	2		40–49	0.85	3
	50–59	1.23	4		50–59	1.79	4
	60–69	1.68	4		60–69	2.75	5
BP, mmHg	<120/ < 80		0	BMI, kg/m^2^	<24		0
	120–129/80–84	0.64	2		≥25	0.35	2
	≥130/ ≥ 85	0.93	3	BP, mmHg	<120/ < 80		0
FBS, mg/dL	<126		0		120–129/80–84	0.76	2
	≥126	0.76	2		≥130/ ≥ 85	0.86	3
TG, mg/dL	<150		0	HR, bpm	<61		0
	≥150	0.19	1		≥61	0.29	2
eGFR, mL/min/1.73 m^2^	eGFR≥60		0				
	eGFR<60	0.47	2				
Habitual drinking, %	No		0				
	Yes	0.24	2				

We assigned each category of risk factor with one of the following point scores, corresponding to the β coeficients of multivariable logistic regression: 1, β = 0.01-0.20; 2, β = 0.21-0.80; 3, β = 0.81-1.20; 4, β = 1.21-2.20; and 5, β > 2.20. The reference category for each variable was given a score of 0. BMI body mass index, *BP* blood pressure, *HR* Heart rate, *FBS* fasting blood sugar, *TG* triglycerides, *eGFR* estimated glomerular filtration rate, β standardized partial regression coefficient

Table 5 shows the predictive performance of the risk score for the 5-year incidence of baPWV ≥ 1400 cm/s using the Youden index. For men, the cutoff point was a risk score of 7 points, which yielded a sensitivity of 0.631 and a specificity of 0.636. For women, the Youden index was the highest at 8 points, with a sensitivity of 0.680 and specificity of 0.320.

**Table 5:** Predictive performance of risk scores for 5-year incidence of baPWV ≥ 1400 cm/s

Men				Women			
Risk score	Sensitivity	Specificity	Youden index	Risk score	Sensitivity	Specificity	Youden index
≥ 1	0.005	1.000	0.005	≥ 2	0.005	0.996	0.004
≥ 2	0.021	0.998	0.019	≥ 3	0.031	0.969	0.024
≥ 3	0.060	0.988	0.048	≥ 4	0.079	0.921	0.050
≥ 4	0.159	0.954	0.113	≥ 5	0.176	0.824	0.115
≥ 5	0.278	0.887	0.165	≥ 6	0.297	0.703	0.172
≥ 6	0.413	0.799	0.213	≥ 7	0.495	0.505	0.279
≥ 7	0.631	0.636	0.266	≥ 8	0.680	0.320	0.329
≥ 8	0.723	0.539	0.262	≥ 9	0.830	0.170	0.260
≥ 9	0.888	0.333	0.221	≥ 10	0.940	0.060	0.242
≥ 10	0.917	0.266	0.183	≥ 11	0.967	0.033	0.162
≥ 11	0.974	0.104	0.078	≥ 12	0.994	0.006	0.058
≥ 12	0.996	0.024	0.020				

**Fig. 2:**
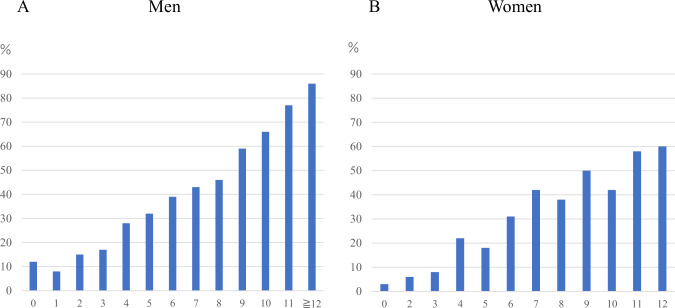
Prevalence of the 5-year occurrence of baPWV ≥1400 cm/s for each score in men and women. **A** men; **B** women

Figure 2 shows the prevalence of baPWV ≥ 1400 cm/s occurring within 5 years for each score in men and women. The prevalence of baPWV ≥ 1400 cm/s occurring increased with increasing scores for both sexes. For scores 0–3, this prevalence was less than 20% in both sexes; for scores 9 and above, the prevalence exceeded 50% in both sexes.

### Equation predicting the 5-year incidence of baPWV ≥ 1400 cm/s in men and women

We developed an equation to predict the probability of baPWV ≥ 1400 cm/s occurring within 5 years, using the risk factors that showed the significant differences in Table 2, such as age (years), BMI (kg/m^2^), SBP (mmHg), DBP (mmHg), HR (bpm), log FBS, log TG in men, and age (years), SBP (mmHg), DBP (mmHg), HR (bpm), and smoking history (0/1) in women, as follows:

$${{\rm{Probability}}}({{\rm{men}}})=\;	 1/(1+{{\rm{Exp}}}[-\{-16.14813+0.06839\\ 	 \times {{\rm{A}}}{{\rm{ge}}}-0.04200\times {{\rm{BMI}}}+0.02589\times {{\rm{SBP}}} \\ 	 +0.01981\times {{\rm{DBP}}}+0.01141\times {{\rm{HR}}} \\ 	 +1.48546\times \log {{\rm{FBS}}}+0.23870\times \log {{\rm{TG}}}\}])$$

$${{\rm{Probability}}}({{\rm{women}}})= 	1/(1+\exp [-\{-12.20559+0.10546 \\ 	 \times {{\rm{Age}}}+0.02980\times {{\rm{SBP}}}+0.01604 \\ 	 \times {{\rm{DBP}}}+0.02040\times {{\rm{HR}}}+0.51429 \\ 	 \times {{\rm{Smoking}}}\}]).$$

The median probability obtained from the cohort was 0.31 [0.19, 0.44] in men and 0.23 [0.13, 0.38] in women. The AUC of the ROC curves for 5-year baPWV ≥ 1400 cm/s in men was 0.71 and 0.77 in women (Fig. 1B). Compared with score-based evaluation methods, the equation evaluation method fared better at discriminating the occurrence or not of baPWV ≥ 1400 cm/s within 5 years in both sexes; however, using it in clinical settings is complicated.

## Discussion

In this study, we examined the factors associated with the 5-year incidence of baPWV ≥ 1400 cm/s in Japan based on large-scale health examination data. The results showed that age, BP, FBS, TG, eGFR, and habitual drinking were factors significantly associated with the 5-year incidence of baPWV ≥ 1400 cm/s in men, whereas age, BMI, BP, and HR were significantly associated with the 5-year incidence of baPWV ≥ 1400 cm/s in women. Using these factors, we developed a risk-prediction score and risk prediction equation for each sex. The risk prediction score based on the abovementioned factors for the 5-year incidence of baPWV ≥ 1400 cm/s yielded an AUC = 0.68 for men and 0.71 for women. Furthermore, the risk prediction equation for the 5-year incidence of baPWV ≥ 1400 cm/s yielded an AUC = 0.71 for men and 0.77 for women, suggesting that the equation had better prediction power than that of the risk prediction score.

Hypertension, dyslipidemia, DM, smoking, obesity, CKD, age, and male sex are risk factors for atherosclerosis [12, 13, 25]. The Framingham risk score (FRS), which was developed for the American population, uses age, sex, SBP, LDL-C, HDL-C, and smoking as risk factors to predict the development of CHD [5]. In contrast, the Suita score, developed for Japanese subjects, includes age, sex, SBP, DBP, LDL-C, HDL-C, DM, smoking, and CKD as risk factors [26].

As an index of arterial stiffness, the simple and non-invasive baPWV has been used in clinical practice and large cohort studies [11]. Previous cross-sectional studies have reported that age, sex, SBP, DBP, HR, FBS, TG, and eGFR levels are significantly associated with elevated baPWV in men and women. Yamashina et al. reported that age, BMI, mean BP, FBS, HDL-C, TG, and smoking were significantly associated with baPWV ≥ 1400 cm/s in both men and women in a Japanese population aged ≥ 30 years.

Previous cross-sectional studies have reported that age is significantly associated with baPWV. In a cross-sectional study of 7881 Japanese health examination participants (mean age, 43 years, 4488 men and 3393 women), Tomiyama et al. reported that aging affected baPWV in both men and women, with the effect greater in women than in men [8]. We previously reported that baPWV was independently correlated with age in 1033 Japanese participants (567 men and 466 women) who underwent regular health examinations [27]. In the present longitudinal study, age was significantly associated with the 5-year incidence of baPWV ≥ 1400 cm/s in both men and women.

Cross-sectional and longitudinal studies have reported an association between BP and baPWV. We previously reported a strong correlation between the mean BP and baPWV in a cross-sectional study of 567 Japanese men and 466 Japanese women undergoing regular health examinations (men: *r* = 0.617, women: *r* = 0.753) [27]. In addition, in a longitudinal study of 1020 Chinese young adults aged 18–23 years (47.7% women) without cardiovascular disease who were followed up for up to 25 years, early stage 1 hypertension (130–139/80–89 mmHg) was compared with normotension in Chinese participants in middle adulthood. The risk of developing high-risk baPWV was reported to be 1.61 times higher in those with early stage 1 hypertension than in normotensive participants [15]. Our present 5-year longitudinal study demonstrated that normal-high BP (120–129/80-84 mmHg) and hypertension (≥ 130/ ≥ 85 mmHg) were significantly associated with a 5-year incidence of baPWV ≥ 1400 cm/s, as compared with normal BP ( < 120/ < 80 mmHg), in both men and women.

Cross-sectional and longitudinal studies have reported an association between HR and baPWV. In a cross-sectional study of 68 men and women (mean age 65.97 ± 9.90) living in China, the results demonstrated that changes in HR may affect the baPWV, and the baPWV values tended to be higher when HR accelerated [28]. In addition, in a longitudinal study of 1795 healthy Japanese individuals (mean age 39 ± 8 years) followed for 5–6 years, HR at the baseline examination and changes in HR during the follow-up period were significantly associated with the corresponding changes in baPWV during the study period [29]. Our 5-year longitudinal study revealed that HR is associated with the 5-year incidence of baPWV ≥ 1400 cm/s in only women.

Cross-sectional and longitudinal studies have reported an association between eGFR and baPWV. In a cross-sectional study of 1,368 men and women (mean age 58.1 ± 14.4 years) living in China, the baPWV values were significantly higher in patients at the CKD stage (eGFR < 60 mL/min/1.73 m^2^) and the early CKD stage (eGFR 60–80 mL/min/1.73 m^2^) [14]. In addition, in a longitudinal study of 8,045 Chinese (mean age 54 ± 12 years) followed for 5 years, participants with higher baPWV at baseline had greater declines in eGFR over time [30]. Our present 5-year longitudinal study revealed that CKD (eGFR < 60 mL/min/1.73 m^2^) is associated with the 5-year incidence of baPWV ≥ 1400 cm/s in men.

An association between the FBS and baPWV has also been reported in cross-sectional studies. A cross-sectional study of 601 drug-naïve healthy participants (men, 46.2%) in Korea reported that FBS was significantly associated with increased baPWV [31]. Another cross-sectional study of 232 men (mean age 65.2 ± 9.5 years) from a rural area in Japan reported that the normal FBS group had a baPWV of 1518 cm/s, whereas the group with diabetes (FBS ≥ 126 mg/dL) had a significantly higher baPWV of 1771 cm/s. Furthermore, multiple regression analyses, including age, SBP, total cholesterol (TC), and BMI, demonstrated that FBS was independently and significantly associated with baPWV in Japanese men [32]. However, the association between FBS and baPWV in longitudinal studies has not been reported. Here, we revealed that elevated FBS ( ≥ 126 mg/dL) is associated with the 5-year incidence of baPWV ≥ 1400 cm/s in men.

Cross-sectional and longitudinal studies have reported an association between lipid levels and baPWV. A cross-sectional study of 909 Chinese participants aged 24–84 years has reported that TG levels were significantly and positively associated with baPWV [33]. In addition, a longitudinal study of 659 Chinese men aged 18 years and older, followed for 4.1 years, examined the association of TC, TG, LDL-C, and HDL-C with elevated baPWV and found that baseline serum TG was independently associated with the incidence of elevated baPWV (baPWV ≥ 1400 m/s) in the normal baPWV (baPWV < 1400 m/s) at baseline [16]. However, their study used only lipid values and did not examine associations with other atherosclerotic risk factors. In our longitudinal study using multiple atherosclerotic risk factors, we found a significant association between TG and the 5-year incidence of baPWV ≥ 1400 m/s in men.

Cross-sectional and longitudinal studies have reported an association between alcohol consumption and baPWV. The baPWV was positively associated with alcohol consumption in Korean men aged 40 years and older, but no clear relationship was found in Korean women in a cross-sectional study [13]. In a longitudinal study of 4016 healthy male Japanese workers (mean age, 43 years), divided into 1306 non-drinkers, 1311 mild-moderate drinkers, and 1337 heavy drinkers, who were followed-up for 9 years, the mean baPWV of non-drinkers was 1306 cm/s, that of mild-moderate drinkers was 1311 cm/s, and that of heavy drinkers was 1337 cm/s, indicating that alcohol intake was significantly associated with elevated baPWV [17]. In our longitudinal study, habitual drinking was associated with the 5-year incidence of baPWV ≥ 1400 cm/s only in men but not in women.

An association between obesity and baPWV has also been reported in cross-sectional studies. In a Chinese study of 429 healthy volunteers (66% men) aged 18 years or older (mean age: 44 years), BMI levels were reported to be significantly associated with baPWV [34]. Furthermore, in a study of 3512 Japanese (1228 men and 2284 women), baPWV was significantly associated with BMI in women with obesity but not in non-obese women and men [35]. In our longitudinal study, BMI was significantly associated with the 5-year incidence of baPWV ≥ 1400 cm/s only in women, whereas this association was absent in men. As shown in Table 1, the percentage of habitual drinking differed significantly between men and women because eating and drinking habits may differ between both sexes.

The present study had some limitations. First, we recruited participants who underwent health examinations at a single facility in Japan and were interested in their health. Therefore, a participant selection bias could not be avoided. Second, our data were retrospectively collected during annual physical examinations, and the subjects were not continuously monitored. Therefore, we could not determine the onset of baPWV ≥ 1400 cm/s, which precluded us from performing a Cox regression analysis to account for time-to-event. Third, we did not assess the use of medications such as antihypertensive, antidiabetic, or antidyslipidemic agents after the initial examination. These medications must have affected subsequent changes in baPWV. Therefore, medication use should be evaluated during the follow-up period in the next study. Fourth, the data were not prospectively collected. In the future, multicenter prospective longitudinal studies should be conducted to examine the relationship between baPWV and risk factors for arterial stiffness. Fifth, prediction scores and equations were not validated. The predictive ability of these scores and equations for arterial stiffness should be validated in future prospective studies.

## Perspectives in Asia

The baPWV has been reported to be a useful index for evaluating arterial stiffness. Longitudinal studies have reported associations between an elevated baPWV and hypertension, HR, TG, eGFR, alcohol consumption, and smoking. However, these longitudinal studies have only examined the association between limited factors and increased baPWV. Our longitudinal study examined the association between multiple risk factors of arterial stiffness and increased baPWV. We developed a prediction score and prediction equation for the progression of arterial stiffness. These score and equation to predict baPWV ≥ 1400 cm/s after 5 years may be useful for early lifestyle modifications and mediations to prevent arterial stiffness among Asians. However, the further multicenter prospective studies should validate these score and equation in Asian countries.

## Conclusion

We examined the association between the 5-year incidence of baPWV ≥ 1400 cm/s and age, BMI, SBP, DBP, HR, HDL-C, LDL-C, TG, FBS, eGFR, habitual exercise, habitual drinking, and smoking history in Japanese men and women aged 30–69 years. In men, age, BP, FBS, TG, eGFR, and habitual drinking were associated with the 5-year incidence of baPWV ≥ 1400 cm/s. In women, age, BMI, BP, and HR contributed to the 5-year incidence of baPWV ≥ 1400 cm/s. Using these factors, we developed a prediction equation and simple prediction score that is easy to use in clinical practice. The usefulness of these equations and prediction score needs to be validated in further prospective studies at multiple centers.
